# Anchor-based bisulfite sequencing determines genome-wide DNA methylation

**DOI:** 10.1038/s42003-022-03543-1

**Published:** 2022-06-16

**Authors:** Nathaniel Chapin, Joseph Fernandez, Jason Poole, Benjamin Delatte

**Affiliations:** Advanced Research Laboratory, Active Motif, 1914 Palomar Oaks Way STE 150, Carlsbad, CA 92008 USA

**Keywords:** Epigenomics, Next-generation sequencing

## Abstract

Whole Genome Bisulfite Sequencing (WGBS) is the current standard for DNA methylation profiling. However, this approach is costly as it requires sequencing coverage over the entire genome. Here we introduce Anchor-Based Bisulfite Sequencing (ABBS). ABBS captures accurate DNA methylation information in *Escherichia coli* and mammals, while requiring up to 10 times fewer sequencing reads than WGBS. ABBS interrogates the entire genome and is not restricted to the CpG islands assayed by methods like Reduced Representation Bisulfite Sequencing (RRBS). The ABBS protocol is simple and can be performed in a single day.

## Abstract (web summary)

Anchor-Based Bisulfite Sequencing (ABBS) is a sequencing based method that enables analysis of DNA methylation states, based on specific hybridization of bisulfite-refractory methylcytosines with 8-aza-7-deaza-2-deoxyguanosine, incorporated in a primer at the end of 5 random nucleotides.

## Introduction

DNA epigenetics refers to the study of chemical adducts that can be attached to DNA as modifications of nucleotides. The most prevalent DNA modification is methylation of the fifth carbon of cytosines (5mC), initially described in 1925 when Johnson and Coghill discovered its existence in nucleic acids isolated from *Tubercle bacillus*^1^. In mammals, cytosine methylation is predominantly found in CpG dinucleotides and is crucial for development^2^. Local and global alterations in methylation profiles are also associated with diseases such as cancer^3^ and neurodegenerative disorders^4^. It is therefore essential to develop methods that can accurately and quantitively measure the abundance of this so-called “fifth base”^5^ genome-wide.

Currently, there are two classes of 5mC profiling methods: (1) base-resolution approaches, which include Whole Genome Bisulfite Sequencing (WGBS)^6^, Reduced Representation Bisulfite Sequencing (RRBS)^7^, and microarray-based approaches (e.g., Infinium)^8^; and (2) non-base resolution methods such as MeDIP-seq (Methylated DNA Immunoprecipitation -sequencing)^9^ and the MIRA (methylated-CpG island recovery assay) protocol^10^. Base-resolution approaches take advantage of the selective conversion of unmethylated cytosines to uridines by sodium bisulfite. Among these, WGBS is the current gold standard as it provides a complete map of 5mC genome-wide. However, its use is restricted by a high cost linked to its enormous sequencing power requirement (typically over 1 billion reads per mammalian genome)^11^. RRBS reduces the amount of reads needed by focusing exclusively on genomic fragments that contain CpG islands (CGIs), but overlooks methylation in non-CGI regions^5,7,12^. Similarly, microarray-based profiling methods interrogate small sections of the genome and provide no information elsewhere. Non-base-resolution methods circumvent the high cost of WGBS by capturing methylated fragments with specific antibodies^9^ or methyl-binding protein complexes^10^. However, the resolution of these approaches is restricted by the size of the DNA fragments (typically ~300 bp) captured. Hence, there is currently a strong demand for a cost-effective procedure that provides detection of methylcytosines genome-wide at base resolution.

Here we present Anchor-Based Bisulfite Sequencing (ABBS), a method that uses anchored oligonucleotides to target methylated regions over the entire genome after bisulfite conversion. ABBS achieves base resolution with a detection accuracy and coverage of methylated sites similar to that of WGBS, while requiring up to 10 times fewer reads.

## Results and discussion

**Fig. 1:**
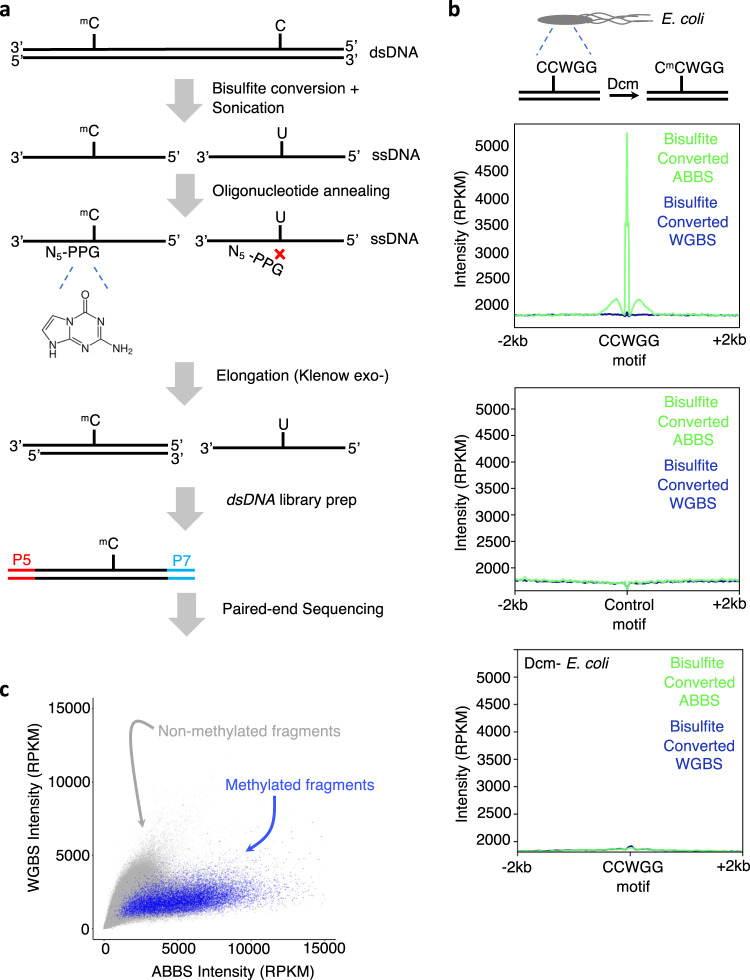
ABBS detects 5mC in *Escherichia coli*. **a** Schematic of the ABBS approach. ^m^C = methylcytosine. **b** ABBS sequencing reads accumulate at the CCWGG motif where methylation is expected. The distributions of ABBS and WGBS signals after bisulfite conversion are shown around the Dcm methylase motif (CCWGG, top) and a control motif (AASTT, middle), or in a *Dcm - E. coli* B strain (bottom). ^m^C = methylcytosine **c** ABBS offers increased coverage of 5mC sites. The WGBS and ABBS coverages are compared for each 10 bp bin over the *E. coli* genome. Methylated fragments are shown in blue and unmethylated fragments are shown in gray.

In the ABBS procedure (Fig. 1a), genomic DNA is treated with Sodium Bisulfite (NaHSO_3_) at high temperature, resulting in the conversion of unmodified cytosines to uridines, while methylated cytosines remain unaffected. The bisulfite acidity combined with high temperatures also induces DNA fragmentation and denaturation into single-stranded DNA. Sonication is then used to generate fragments between 200 and 300 nucleotides in length. Next, methylated regions are targeted with an anchored primer that contains five random nucleotides followed by a 3′ anchor in the form of 8-aza-7-deaza-2-deoxyguanosine (PPG), a pyrazolo[3,4-d]pyrimidine analog that stabilizes PPG:C base-pairing relative to canonical guanosines^13,14^. The anchored primer will incur perfect 3′ hybridization at cytosines that remain after bisulfite treatment (i.e., that were methylated), as bisulfite-refractory methylated cytosines uniquely offer base complementary to the 3′ PPG anchor allowing subsequent elongation with Klenow (exo minus) polymerase. Of note, PPG does not prefer C or 5mC but shows greater affinity than G. By contrast, bisulfite conversion prevents PPG base-pairing at unmethylated cytosines as they are converted to uridines. Standard dsDNA library preparation procedures then allow amplification and sequencing of methylated regions. Notably, while the anchor nucleotide specifically targets methylated sites, the body of the sequencing reads provides an accurate measurement of surrounding methylated and unmethylated cytosines.

We compared the ability of ABBS to identify 5mC genome-wide with the current gold standard, WGBS. For this, we first used the *Escherichia coli* (K12 strain) model, in which the Dcm methylase methylates the second cytosine at CCWGG sites^15,16^. We performed ABBS using the anchored primer described above, and WGBS using fully randomized hexamers (without PPG). In the ABBS sample, the C^m^CWGG motif was directly seen on the aggregate sequences at the beginning of read 1. By contrast, no similar enrichment was observed with WGBS (Sup. Fig. 1a). Notably, bisulfite conversion was efficient (≥99.5%)^17,18^ in all samples, as measured with unmethylated Lambda DNA conversion (see methods). In addition, meta-analyses revealed strong accumulation of ABBS—but not WGBS—reads around genomic occurrences of the Dcm motif (Fig. 1b). By contrast, there was no signal accumulation in a *dcm- E. coli* strain (B strain), around a control motif, nor in samples that were not treated with sodium bisulfite (Fig. 1b middle and bottom panels, Sup. Fig. 1b). These results suggest that the anchored primer efficiently targets the expected C^m^CWGG sites in this model.

We also compared the signal intensities of ABBS and WGBS in methylated and unmethylated regions. For this, we univocally determined methylation status genome-wide using Bismark^19^, and called cytosines with a methylation level >50% as methylated, whereas all other cytosines were called as unmethylated. We then analyzed the signal distribution obtained from both methods for each group. Locally, ABBS reads tend to accumulate predominantly around methylated regions, whereas WGBS reads are spread more evenly (Sup. Fig. 1c). Notably, ABBS signal is abrogated in *dcm-* bacteria (Sup. Fig. 1c). Globally, coverage of non-methylated fragments was comparable—though slightly higher in WGBS—for both methods, while methylated portions of the genome showed increased coverage with ABBS (Fig. 1c), an effect that was absent in the *dcm-* strain (Sup. Fig. 1d). This increased coverage is reproducible between biological replicates (PCC = 0.97, Sup. Fig. 1e) with minimal effects of the NGS library preparation method (New England Biolabs *vs* Active Motif) (Sup. Fig. 1f). These results indicate that the anchored primer successfully redirects sequencing power to methylated regions in ABBS, whereas the WGBS signal is spread more homogeneously across the genome.

**Fig. 2:**
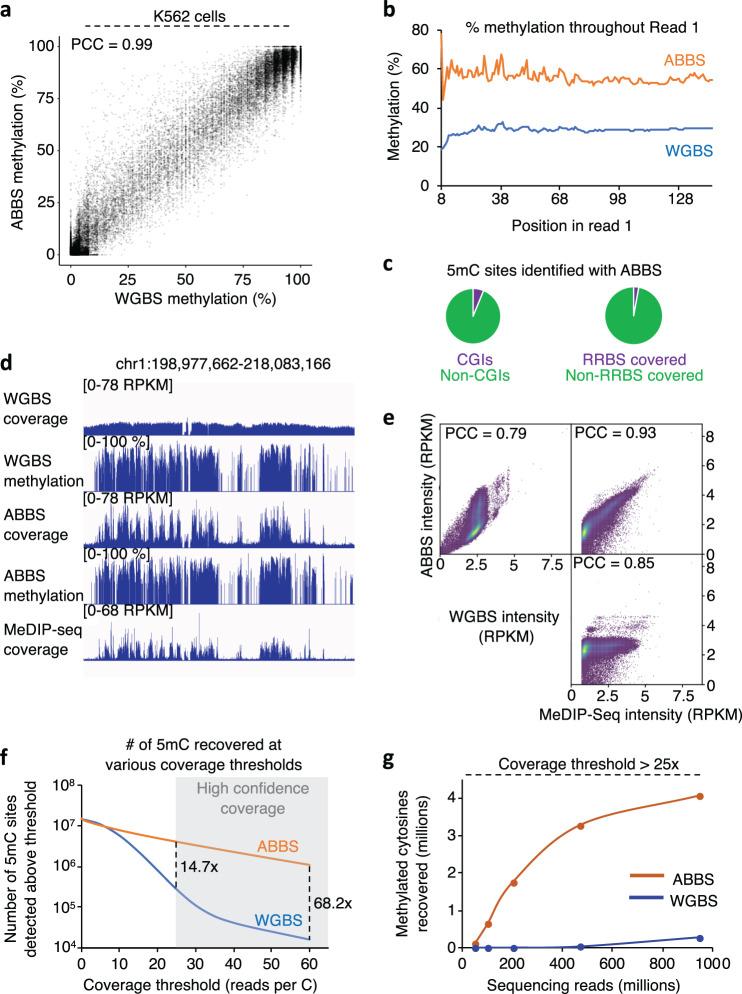
ABBS detects 5mC in human K562 cells. **a** ABBS accurately measures cytosine methylation levels. Scatter plot of the methylation levels measured by WGBS and ABBS for all cytosines with a coverage over 25× in both samples. PCC, Pearson’s correlation coefficient with two-tailed *P* < 2.2 × 10^−16^. **b** ABBS reads contain more 5mC than WGBS reads. The average prevalence of 5mC is shown for ABBS and WGBS at each position in read 1. **c** 5mC sites identified with ABBS are predominantly outside of CGIs and are not covered by RRBS. Shown are the fractions of 5mC sites identified with ABBS (100 million reads subsample, see methods) found inside or outside of CGIs (left), and covered or not covered by RRBS (right). **d** ABBS signals colocalize with MeDIP-seq coverage and with the presence of 5mC. Genome browser tracks show signal distributions for WGBS, ABBS, and MeDIP-seq, as well as methylation levels determined by WGBS and ABBS. **e** ABBS signals colocalize with MeDIP-seq but not WGBS. The coverages for WGBS, ABBS, and MeDIP-seq are compared for each 10-kb bin over the human genome. PCC, Pearson’s correlation coefficient with two-tailed *P* < 2.2 × 10^−16^. **f** ABBS offers increased coverage at 5mC sites, relative to WGBS. Shown are the absolute numbers of 5mC sites (>50% methylation) identified with ABBS (orange) and WGBS (blue), at varying coverage thresholds. **g** The sensitivity of ABBS surpasses that of WGBS for detection of 5mC. Shown are the absolute numbers of 5mC sites (>50% methylation) identified with ABBS (orange) and WGBS (blue), at varying total read counts and with a coverage threshold set at >25×.

Next, we sought to assess the ability of ABBS to accurately measure cytosine methylation in mammalian cells. For this, we performed ABBS and WGBS with human myelogenous leukemia cells (K562, tier 1, ENCODE). Bisulfite conversion was efficient (≥99.5%) for all samples, and the ABBS data were reproducible with two library preparation methods (Sup. Fig. 2a). In the ABBS data, we found a strong enrichment of cytosines preceding the PPG anchor fixed at the 6th position in read 1, consistent with the prevalence of cytosine methylation at CpG dinucleotides in mammals. By contrast, no such enrichment was observed with WGBS (Sup. Fig. 2b). As expected, both methods also detected few occurrences of non-CpG methylation and showed a bimodal distribution of methylation levels^20^ (Fig. 2a, Sup. Fig. 2c). Importantly, there was a strong agreement in the methylation levels measured by ABBS and WGBS (Pearson Correlation Coefficient, PCC = 0.99, Fig. 2a). We also note that ABBS produces slight overestimates relative to WGBS at several cytosines (Sup. Fig. 2d, see points above the diagonal). Notably, the bi-modal distribution of methylation in the human genome results in abundant extreme values (very low and very high methylation levels), which can skew the correlation values. Therefore, we assessed whether ABBS produces accurate measurements at sites of intermediate methylation levels (i.e., between 20% and 80% methylation). At these sites, we found good but decreased correlation values (PCC = 0. 82, Sup. Fig. 2d), which may be due in part to the effect of restricted range on correlation. To circumvent this issue, we also re-calculated the Pearson correlation coefficient in a subset where cytosines are homogeneously distributed on the X-axis, by sampling equal amounts (5000) of cytosines in each of the twenty 5%-wide bins spanning the full range of methylation (i.e., 5000 cytosines in the 0–5% range, 5000 cytosines in the 5–10% range,…, 5000 cytosines in the 95–100% range). In this homogeneously distributed dataset, the methylation levels measured by ABBS strongly correlated with those measured by WGBS (PCC = 0.93, including hypo- and hypermethylated sites, Supp. Fig. 2d, right panel).

To assess the ability of ABBS to direct sequencing power to methylated regions in mammalian cells, we looked at the average methylation rates across sequencing reads and found that methylation levels were about twice as high as in ABBS reads relative to WGBS (Fig. 2b). This is likely due to direct targeting of the anchored primer to 5mC sites combined with the clustering of 5mC in methylation-rich regions (e.g., CpG islands^21^). Notably, ABBS can detect 5mC both inside and outside of CGIs, whereas other broadly used approaches, such as RRBS, are biased toward CGIs (Fig. 2c). We also included a comparison with MeDIP-seq, a non-base-resolution and enrichment-based approach, using published data from the same cell line^22^. ABBS and MeDIP-seq signals aggregate together (PCC = 0.93), typically in 5mC-rich regions, whereas WGBS profiles are more homogeneously distributed across the genome (Fig. 2d, e, Sup. Fig. 2e). We next looked at the signal distribution for each technique on genomic elements. Cytosine methylation is typically depleted at active promoters where it can operate as a repressive epigenetic mark, and is enriched in the gene bodies of actively transcribed genes^23^. A metagene analysis highlighted this specific pattern with ABBS and MeDIP-seq, but not WGBS (Sup. Fig. 2f). Together, these results confirm the ability of the anchor-based approach to selectively target methylated sections of the genome to an extent similar to MeDIP-seq. ABBS also provides a broader view of the methylation landscape than more restrictive approaches like RRBS.

Next, we compared the sensitivity of ABBS and WGBS. For this, we calculated the number of 5mC (>50% methylation level) identified by ABBS and WGBS, when different coverage requirements are used (Fig. 2f). At a coverage over 25×, which represents high confidence measurements^24^, ABBS detects ~15 times more methylated sites than WGBS. ABBS also allows the use of increased coverage—and confidence—cutoffs for which the sensitivity of WGBS drops dramatically (Fig. 2f, grayed area). Due its high sensitivity, we hypothesized that ABBS would require much less sequencing power to achieve results similar to WGBS. To assess this possibility, we subsampled sequencing reads in the ABBS and WGBS datasets and calculated the amount of 5mC recovered when varying total read counts. As expected, detection of 5mC was consistently much more efficient with ABBS (Fig. 2g, Sup. Fig. 2g), with an optimal sensitivity at 200 million ABBS reads (Sup. Fig. 2g). Strikingly, 100 million ABBS reads were sufficient to detect over twice as many 5mC sites as ~1 billion WGBS reads (Fig. 2g). Hence, ABBS can be used to reduce the sequencing power needed for genome-wide 5mC mapping up to tenfold, while maintaining superior sensitivity relative to WGBS.

In this article, we present ABBS as a method for 5mC mapping genome-wide. ABBS provides base resolution detection and is not restricted to predetermined genomic segments, as is the case with other targeted methods. While one weakness of ABBS is calculation of methylation in hypomethylated regions due to limited coverage, the method maintains an accuracy on par with the current gold standard, WGBS, while offering an improved sensitivity at methylated regions that can be used to drastically decrease the required sequencing power from ~1 billion WGBS reads to ~100 million ABBS reads per mammalian genome. Hence, ABBS represents a strong alternative for a cost-effective and base-resolution detection of methylcytosines genome-wide.

## Methods

### Cell culture

*Escherichia coli* K12 cells were acquired from New England Biolabs (NEB, #E4104). Two clones were selected by streaking on LB agar, and cells were grown at 37 °C in liquid LB on a shaker. Optical density was measured at 600 nm every twenty minutes until bacteria reached a plateau phase. The culture was then quickly placed on ice and spun down at 7500 RPM at 4 °C for 10 min, then washed twice with ice cold 1× PBS. Aliquots were snap frozen in liquid nitrogen and kept at −80 °C before genomic DNA extraction. *Escherichia coli* strain B (*dcm-*) genomic DNA was obtained from Sigma (D2001-5MG).

K562 cells (ATCC) were grown in RPMI-1640 supplemented with 2mM L-glutamine, 10% FBS and 50 u/mL penicillin G + 50 µg/mL streptomycin, at 37 C with 5% CO_2_. Subculture was performed following ATCC instructions. Cell preparation for genomic DNA extraction was performed as follows. Cells were trypsinized (ThermoFisher) and counted using a hemocytometer, and viability was assessed with Trypan Blue (ThermoFisher) staining. The culture was then centrifugated at 3000 RPM at 4 °C for 5 min, washed twice with ice cold 1× PBS. Aliquots were then snap frozen in liquid nitrogen and kept at −80 °C before genomic DNA extraction.

### ABBS protocol

Genomic DNA was extracted using the DNeasy Blood & Tissue Kit (Qiagen) following the manufacturer’s protocol. 3 μg of *E. coli* or 10 μg of K562 genomic DNA was spiked with 1% unmethylated Lambda DNA, followed by bisulfite conversion with the Zymo Lightning kit (Zymo Research) following the manufacturer’s instructions, then eluted twice in 10 μl of Tris-HCl 10 mM pH= 8.0 (desulfonation was performed for 20 mins, on column). Genomic DNA was then fragmented by sonication for 15 min on a PIXUL Multisample sonicator (Active Motif, default settings). Samples were then heated at 95 °C for 3 min, then quickly placed on ice. Quantification was done with a Nanodrop (Thermo Fisher) and profiles were measured with an RNA TapeStation (Agilent) to assess sonication efficiency. For control samples that did not undergo bisulfite conversion, genomic DNA was instead sonicated for 30 min using PIXUL Multisample sonicator (default settings) before heating at 95 °C for 3 min, then quickly placed on ice. Samples were then processed further, or stored at −80 °C.

Primed DNA synthesis was performed as follows. 100 ng of bisulfite-treated and sonicated genomic DNA was diluted in 1× NEBuffer 2 (New England Biolabs), supplemented with 0.25 mM dNTPs and 6.5 μM (K562 cells) or 0.65 μM (*E. coli* K12/B strain) ABBS primers (5′-NNNNN-PPG-3′, Integrated DNA Technologies) or WGBS primers (5′-NNNNNN-3′, Integrated DNA Technologies) in 29 μl, heated at 94 °C for 2 min, then rapidly placed on ice for 3–5 min. 1 μl of 5 units/μl Klenow exo minus (New England Biolabs) was added to the reaction, and the temperature was ramped up from 4 °C to 37 °C at a rate of 0.1 °C/s for optimal annealing. Samples were then incubated for 30 min at 37 °C. Free primers were eliminated with 45 μl of AMPure XP beads (Beckman Coulter), following the manufacturer’s instructions. DNA fragments were eluted in 20 μl 10 mM Tris-HCl pH= 8.0 supplemented with 0.05 % Tween-20, and quantified with Qubit 2.0 dsDNA HS kit (Thermo Fisher Scientific).

Double-stranded library preparation was performed with the NEBNext^®^ Ultra™ II DNA Library Prep Kit for Illumina (New England Biolabs) in all samples unless specified otherwise. For the samples labeled “A.M” or “Active Motif”, the Next Gen DNA Library Kit (Active Motif) was used, following manufacturers’ recommendations. Library amplification was performed using KAPA HiFi HotStart Real-Time Library Amp Kit (Roche). After a final purification step using 0.75 volumes of AMPure XP beads, libraries were resuspended in 12 μl of Tris-HCl 10 mM pH= 8.0 supplemented with 0.05 % Tween-20. NGS libraries were quantified with a Qubit 2.0 dsDNA HS kit and profiles were measured with HS dsDNA TapeStation. NGS libraries were then pooled and paired-end sequenced on a NovaSeq (K562) or NextSeq (*E. coli*) system (Illumina).

See Table 1 for sequencing statistics.

**Table 1:** Sequencing statistics.

Sample description	Sequenced reads	Sequencing mode
*E. coli*, K12 strain, clone 1, untreated, WGBS primer	22,591,505	2 × 38
*E. coli*, K12 strain, clone 1, untreated, ABBS primer	18,764,442	2 × 38
*E. coli*, K12 strain, clone 1, bisulfited, WGBS primer	26,900,953	2 × 38
*E. coli*, K12 strain, clone 1, bisulfited, ABBS primer	23,732,818	2 × 38
*E. coli*, K12 strain, clone 2, untreated, WGBS primer	19,196,910	2 × 38
*E. coli*, K12 strain, clone 2, untreated, ABBS primer	18,024,053	2 × 38
*E. coli*, K12 strain, clone 2, bisulfited, WGBS primer	25,126,669	2 × 38
*E. coli*, K12 strain, clone 2, bisulfited, ABBS primer	24,355,597	2 × 38
*E. coli*, B strain (dcm-), bisulfited, WGBS primer	14,661,263	2 × 38
*E. coli*, B strain (dcm-), bisulfited, ABBS primer	15,394,170	2 × 38
*E. coli*, K12 strain, clone 1, bisulfited, ABBS primer (A.M. library prep)	4,593,447	2 × 38
*E. coli*, K12 strain, clone 2, bisulfited, ABBS primer (A.M. library prep)	2,843,166	2 × 38
K562, WGBS experiment	928,115,345	2 × 150
K562, ABBS experiment	927,040,524	2 × 150
K562, ABBS experiment (A.M. library prep)	274,947,517	2 × 38

### Bioinformatic analyses

Raw reads were aligned to the human hg19 or *E. coli* K12 genomes using Bismark (https://github.com/FelixKrueger/Bismark) [command: $ bismark <genome path> -p 4 --parallel 6 -q --pbat -o <output folder> -1 read1.fastq.gz -2 read2.fastq.gz], and PCR duplicates were removed [command: $ deduplicate_bismark -p --output_dir <output folder> --bam <input bam file>]. A MAPQ filter of 20 was then applied using samtools (https://github.com/samtools/samtools) [command: $ samtools view -h -q 20 <input bam file > -o <output folder bam file>]. Cytosine methylation was measured with Bismark Methylation Extractor [command: $ bismark_methylation_extractor --parallel 10 -p --no_overlap --ignore 7 --ignore_r2 7 --ignore_3prime 5 --ignore_3prime_r2 5 -o <output folder> --report --bedGraph --zero_based --cutoff 1 --CX --remove_spaces --cytosine_report --genome_folder <genome path> <input bam file>]. Next, bam files were sorted and indexed using samtools sort and samtools index. Bigwig coverage files were obtained with deeptools bamcoverage (https://github.com/deeptools/deepTools) [command: $ bamCoverage -b <input bam file> -o <output folder bigwig file> --normalizeUsing RPKM --binSize 5 --numberOfProcessors 16]. The last two columns of the Bismark “*zero coverage file*” were summed using [command: $ awk ‘BEGIN{IFS = “\t”}{$5 = $5 + $6;print $1”\t” $2”\t” $3”\t” $4”\t” $5”\t”}’ bismark.zero.cov > bismark.zero.cov.sum.bedgraph]. Chromosome M and non-canonical chromosomes were also removed from the bedgraph files. Cytosines with methylation levels above 50% were then called as methylated [command: $ awk ‘{ if ($4 > 50) { print } }’ <Input bedgraph> > <output bedgraph>]. Cytosines with a coverage above a minimum cutoff were also selected [command: $ awk ‘{ if ($5 > coverage) { print } }’ <Input bedgraph> > <output bedgraph>]. Bam files subsampling was achieved with sabamba (https://lomereiter.github.io/sambamba/) [command: $ sambamba view -h -t 20 -s 0.5 -f bam --subsampling-seed=variable <Input bam file>  -o <output bam file>]. Meta-analyses and metagene analysis were generated using Galaxy interface (https://usegalaxy.org) with computeMatrix and plotHeatmap with defaults parameters (except conversion of missing values to zero). Scatter plots were generated using Galaxy interface with multiBigwigSummary and plotCorrelation (Fig. 2e), or multiBigwigSummary and RStudio (all other scatter plots), with defaults parameters. CG, CHG and CHH methylation distributions were produced with ViewBS (https://github.com/xie186/ViewBS). Overlaps in Fig. 2c were calculated with Galaxy Intersect, with 1 bp overlap.

### Statistics and reproducibility

All PCC, Pearson’s correlation coefficient, were calculated with a two-tailed test.

### Reporting summary

Further information on research design is available in the Nature Research Reporting Summary linked to this article.

## Supplementary information

Peer Review File

Supplementary information

Description of Additional Supplementary Files

Supplementary Data 1

Reporting Summary

## Data Availability

ABBS and WGBS sequencing data can be found on GEO (GSE180796). MedIP-seq data can be found on GEO (GSM1368906). RRBS data was downloaded from ENCODE (http://hgdownload.cse.ucsc.edu/goldenPath/hg19/encodeDCC/wgEncodeHaibMethylRrbs/). Select the file wgEncodeHaibMethylRrbsH1hescHaibSitesRep1.bed.gz. The source data for graphs prepared for the manuscript are available as Supplementary Data 1.
