# Supplementary material for: Anchor-based bisulfite sequencing determines genome-wide DNA methylation
Source: Commun Biol. 2022 Jun 16;5:596. doi: 10.1038/s42003-022-03543-1 (PMC9203462; doi:10.1038/s42003-022-03543-1)
Supplement: Supplementary file 2 — Supplementary information [file 42003_2022_3543_MOESM2_ESM.pdf]

Supplementary Information

Anchor-Based Bisulfite Sequencing determines genome-wide DNA methylation

Nathaniel Chapin[1], Joseph Fernandez[1], Jason Poole[1], Benjamin Delatte[1*]

[1] Advanced research laboratory, Active Motif, 1914 Palomar Oaks Way STE 150, Carlsbad, CA 92008, USA.

*To whom correspondence should be addressed. Email: bdelatte@activemotif.com

Supplementary Figure 1a

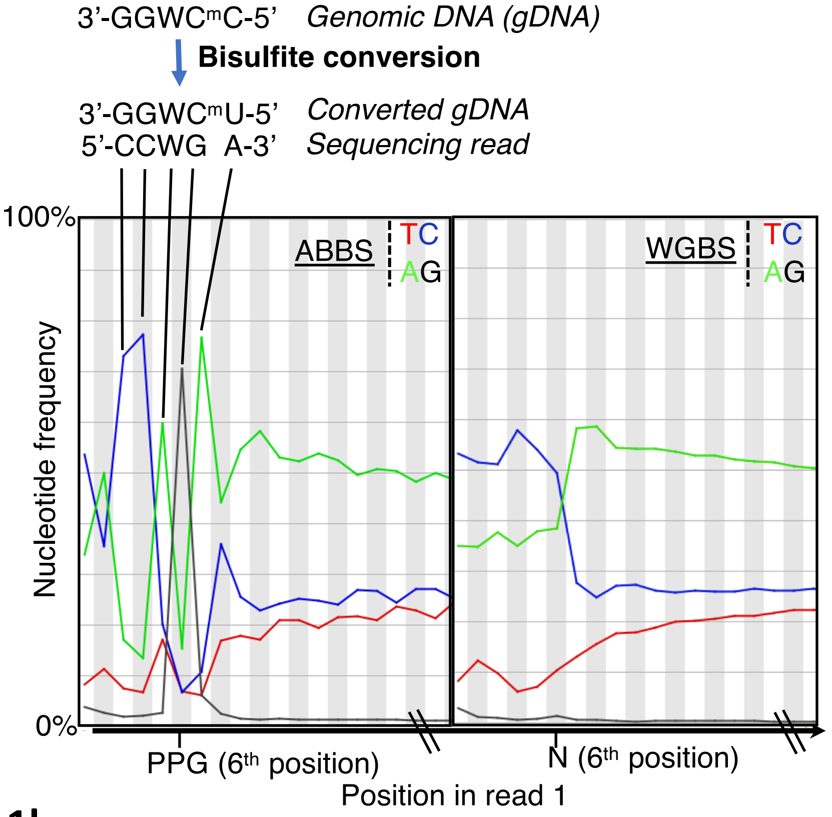

1b

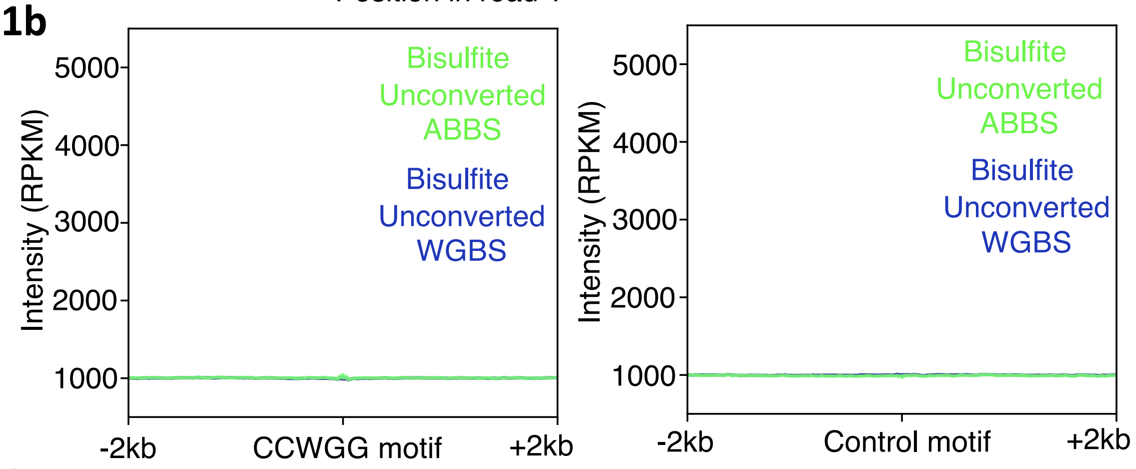

1c

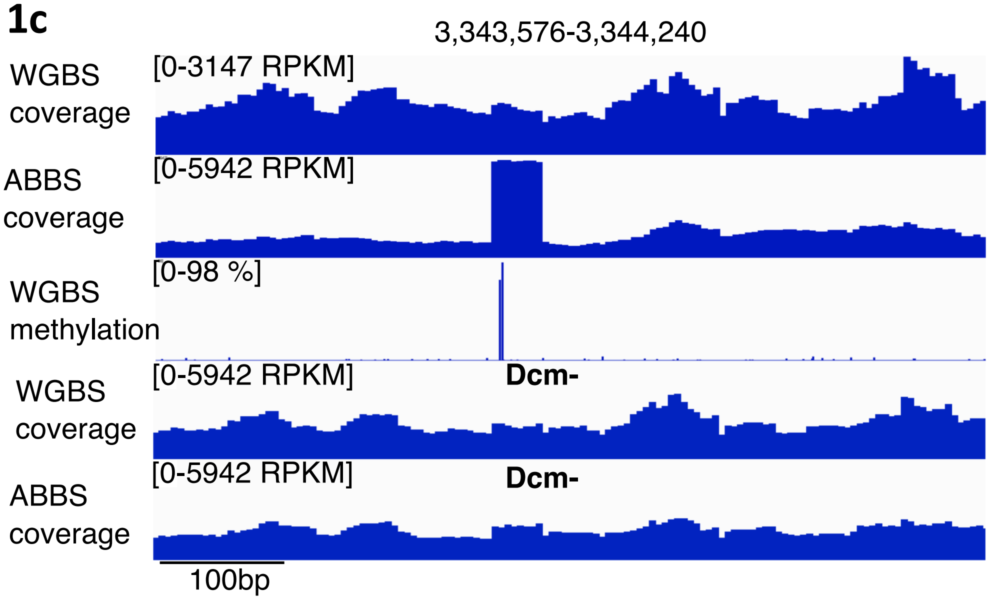

Supplementary Figure 1d

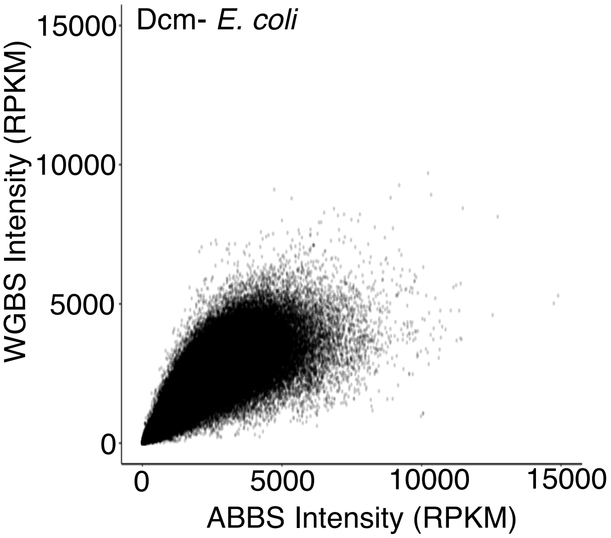

1e

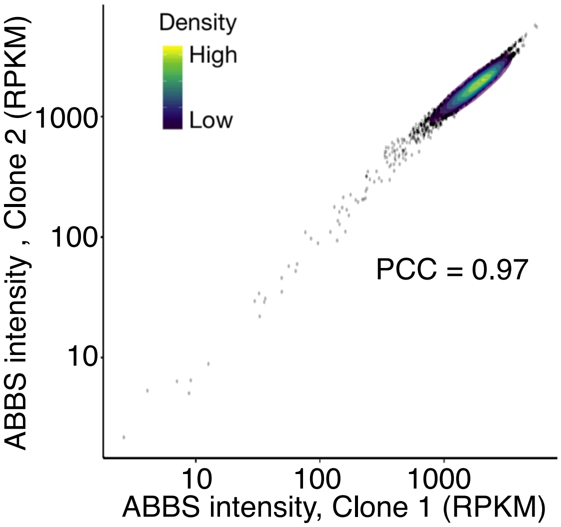

1f

Clone 1

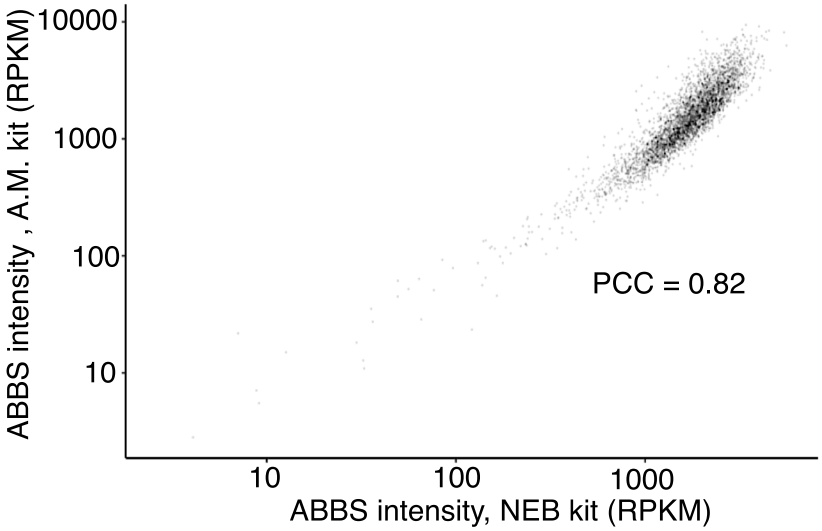

Clone 2

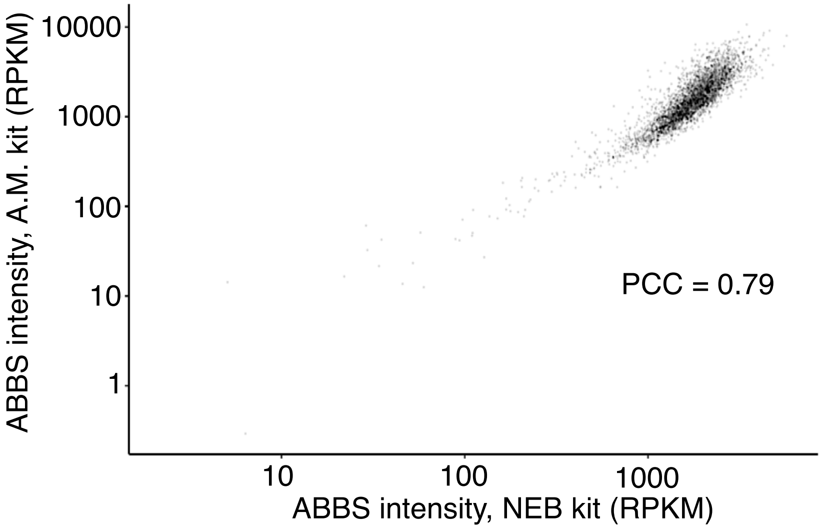

Supplementary Figure 1. **ABBS detection of 5mC in *Escherichia coli*.**

a, The Dcm motif is directly seen in read 1 in ABBS samples. FastQC analysis depicting the aggregate base composition of read 1 for ABBS and WGBS. gDNA = genomic DNA. C^m= methylcytosine. **b**, ABBS reads are evenly distributed in the absence of bisulfite treatment. Meta-analyses of the CCWGG methylation motif (top) and a control motif (AASTT, bottom) in *Escherichia coli* (K12 strain). Unconverted = sodium bisulfite treatment omitted in the sample preparation. **c**, ABBS but not WGBS reads accumulate at 5mC sites. Genome browser tracks showing WGBS and ABBS coverages, as well as methylation called by Bismark on the WGBS sample. Dcm- B strain *E. coli* is also shown. **d**, The WGBS and ABBS coverages are compared for each 10 bp bin over the *E. coli* genome that does not contain dcm methyltransferase. **e**, ABBS coverage is reproducible. Scatter plot of the ABBS coverage (1-kb bins) in two *E. coli* K12 clones is shown. PCC, Pearson's correlation coefficient with two-tailed $P < 2.2 \times 10^{-16}$. **f**, ABBS is reproducible with two library preparation methods. Scatter plots of the ABBS coverage signals (1-kb bins) in two *E. coli* K12 clones, obtained with two different library preparation methods (New England Biolabs vs Active Motif) are shown. PCC, Pearson's correlation coefficient with two-tailed $P < 2.2 \times 10^{-16}$. A.M. = Active Motif. NEB = New England Biolabs.

Supplementary Figure 2a

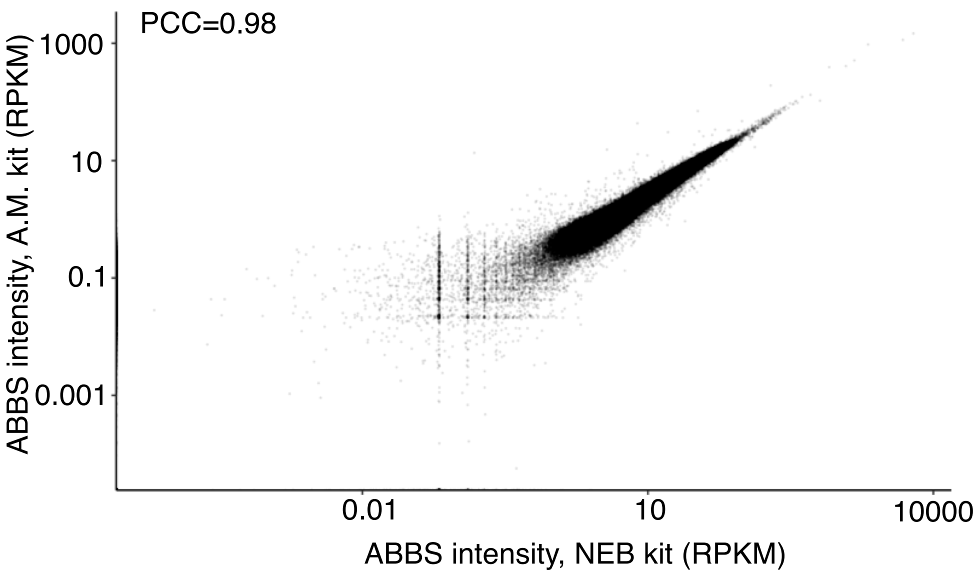

2b

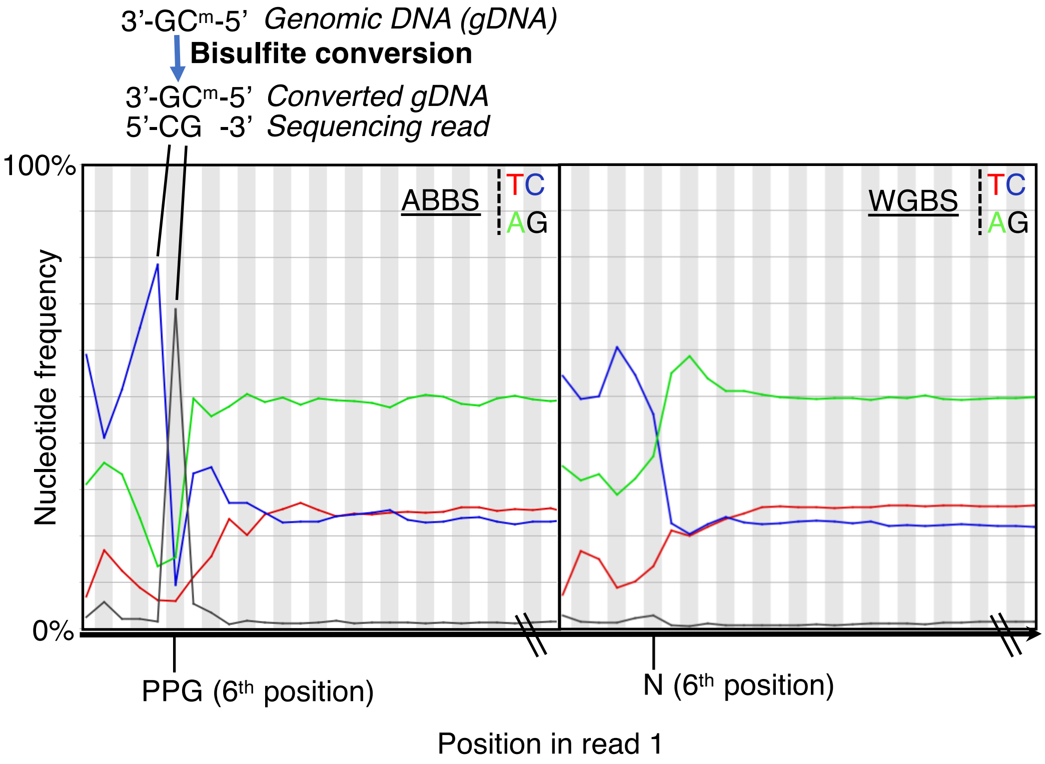

2c

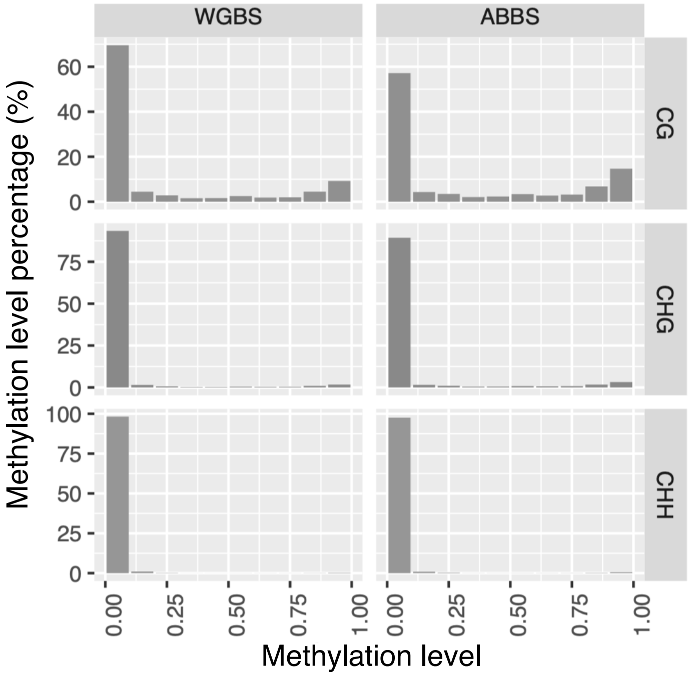
WGBS
ABBS
CG
CHG
CHH
Methylation level percentage (%)
Methylation level
0.00
0.25
0.50
0.75
1.00

2d

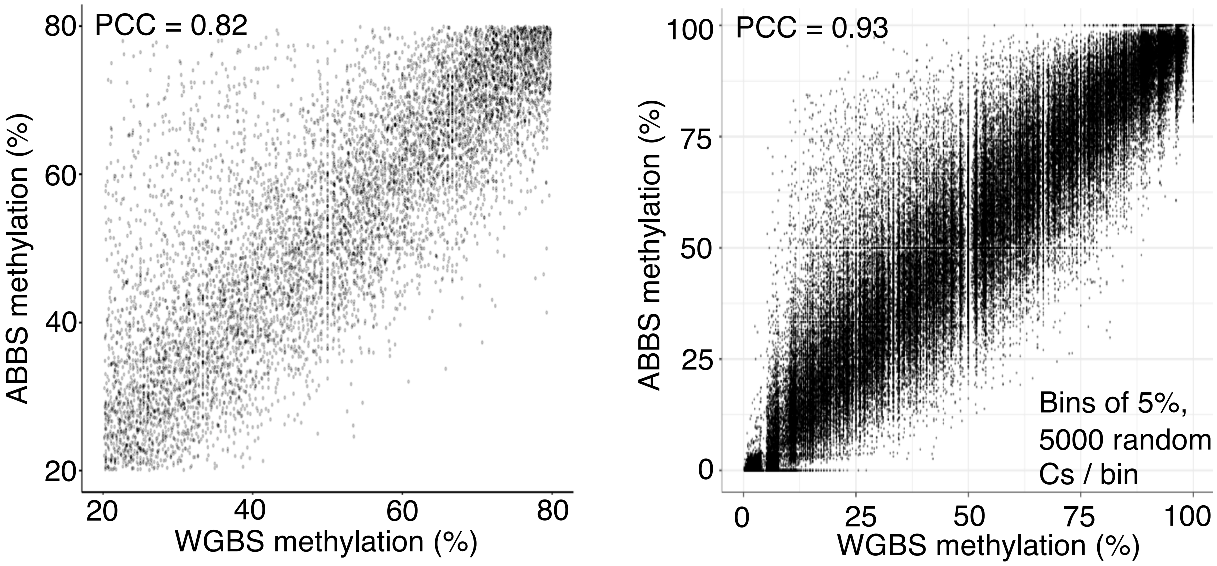
PCC = 0.82
ABBS methylation (%)
WGBS methylation (%)
PCC = 0.93
Bins of 5%,
5000 random
Cs / bin

2e

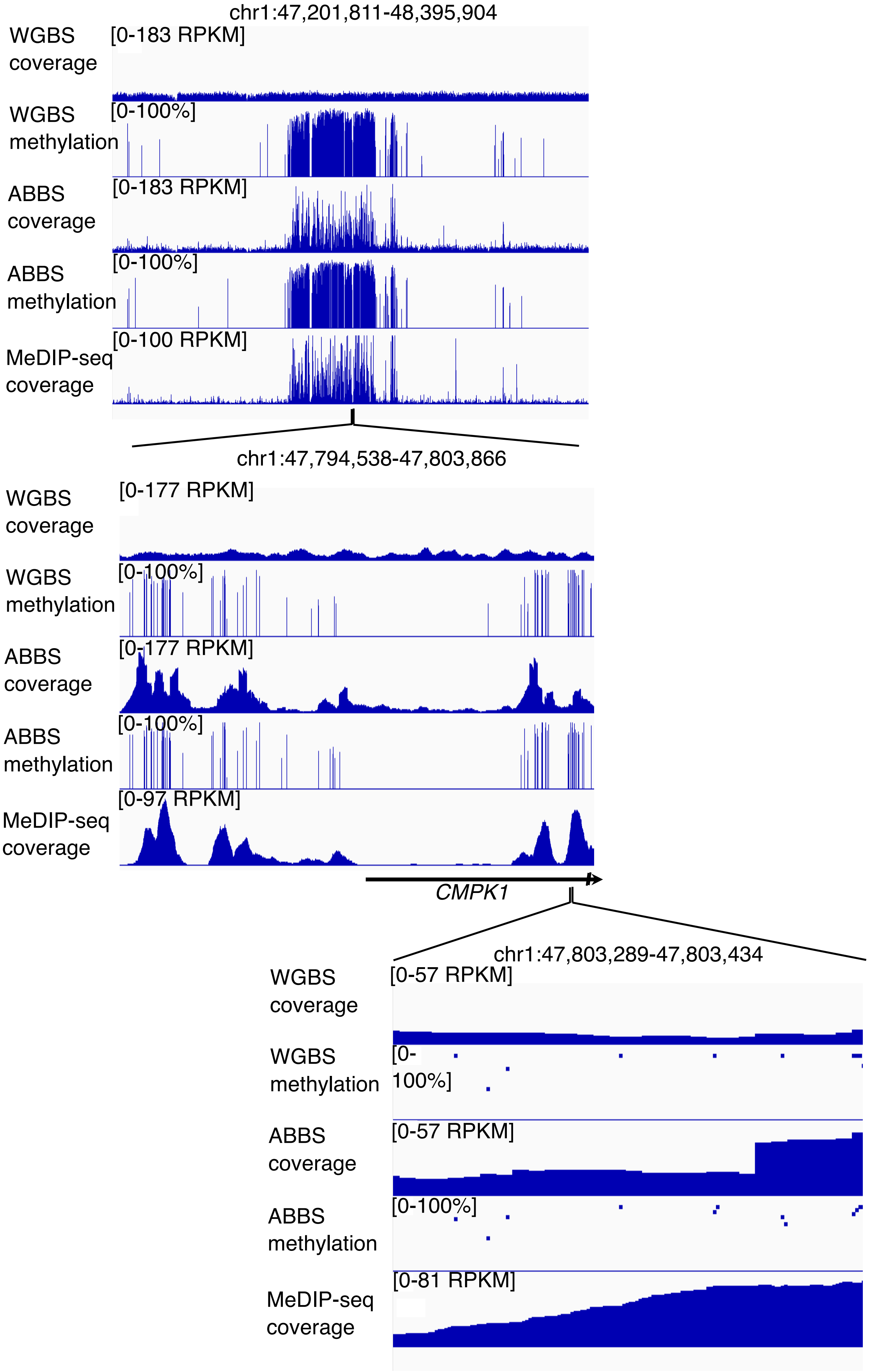
chr1:47,201,811-48,395,904
WGBS coverage
[0-183 RPKM]
WGBS methylation
[0-100%]
ABBS coverage
[0-183 RPKM]
ABBS methylation
[0-100%]
MeDIP-seq coverage
[0-100 RPKM]
chr1:47,794,538-47,803,866
WGBS coverage
[0-177 RPKM]
WGBS methylation
[0-100%]
ABBS coverage
[0-177 RPKM]
ABBS methylation
[0-100%]
MeDIP-seq coverage
[0-97 RPKM]
CMPK1
chr1:47,803,289-47,803,434
WGBS coverage
[0-57 RPKM]
WGBS methylation
[0-100%]
ABBS coverage
[0-57 RPKM]
ABBS methylation
[0-100%]
MeDIP-seq coverage
[0-81 RPKM]

(2e)

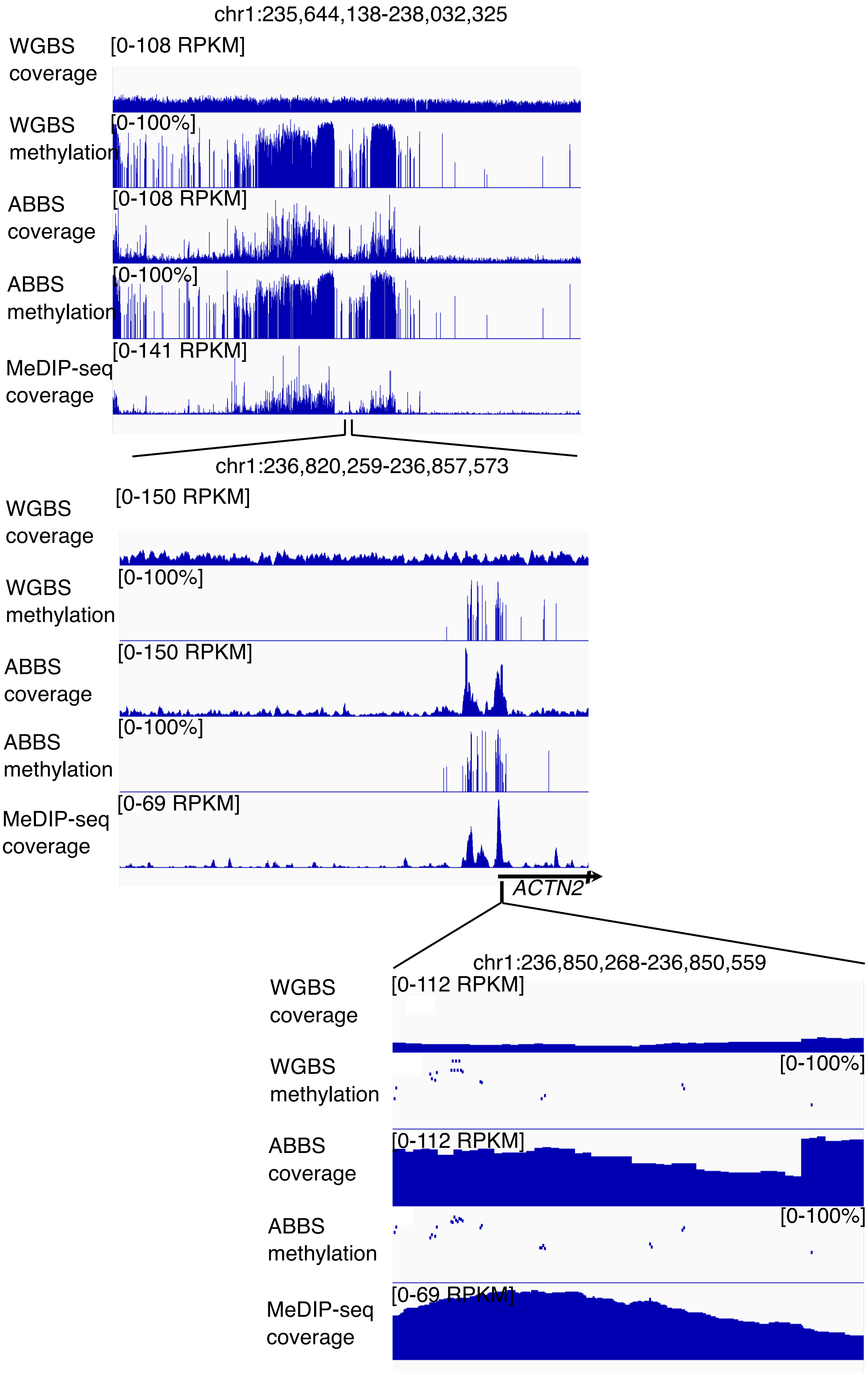
chr1:235,644,138-238,032,325
WGBS coverage
[0-108 RPKM]
WGBS methylation
[0-100%]
ABBS coverage
[0-108 RPKM]
ABBS methylation
[0-100%]
MeDIP-seq coverage
[0-141 RPKM]
chr1:236,820,259-236,857,573
WGBS coverage
[0-150 RPKM]
WGBS methylation
[0-100%]
ABBS coverage
[0-150 RPKM]
ABBS methylation
[0-100%]
MeDIP-seq coverage
[0-69 RPKM]
ACTN2
chr1:236,850,268-236,850,559
WGBS coverage
[0-112 RPKM]
WGBS methylation
[0-100%]
ABBS coverage
[0-112 RPKM]
ABBS methylation
[0-100%]
MeDIP-seq coverage
[0-69 RPKM]

2f

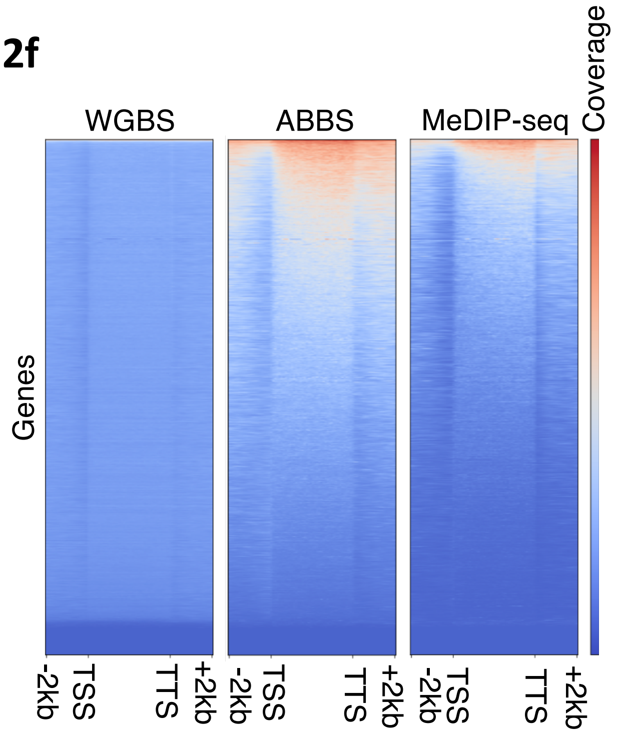
WGBS
ABBS
MeDIP-seq
Coverage
Genes
-2kb
TSS
TTS
+2kb

2g

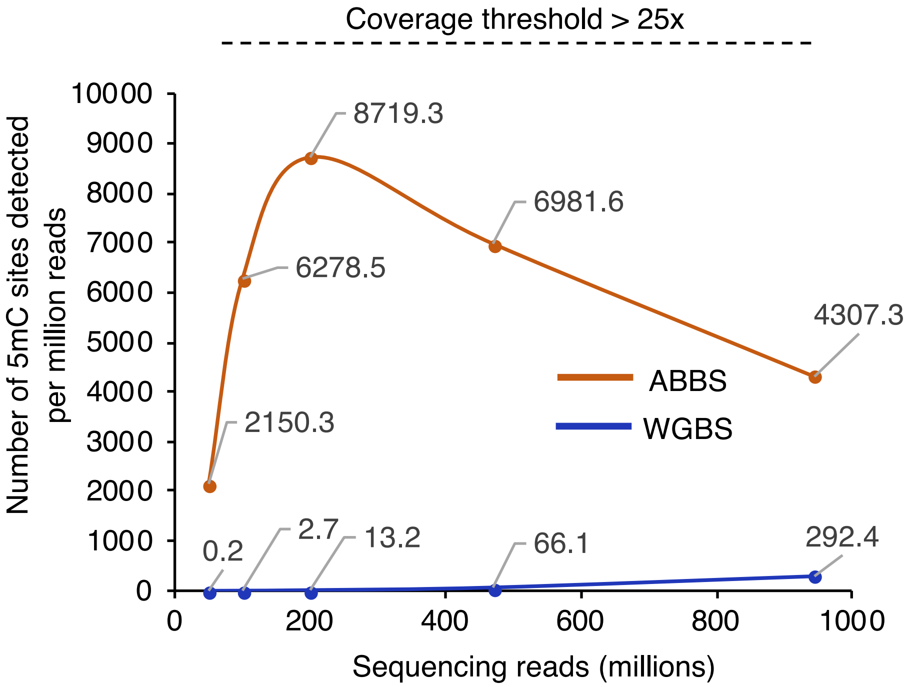
Coverage threshold > 25x
Number of 5mC sites detected per million reads
Sequencing reads (millions)
ABBS
WGBS
8719.3
6981.6
6278.5
4307.3
2150.3
0.2
2.7
13.2
66.1
292.4
0
200
400
600
800
1000
1000
2000
3000
4000
5000
6000
7000
8000
9000
10000

Supplementary Figure 2. **ABBS detection of 5mC in K562 cells.**

a, ABBS coverage directed at 5mC sites is reproducible. Scatter plot of ABBS coverage signals (10-kb bins) obtained with two different library preparation methods (New England Biolabs vs Active Motif) is shown. PCC, Pearson's correlation coefficient with two-tailed $P < 2.2 \times 10^{-16}$. A.M. = Active Motif. NEB = New England Biolabs. **b**, A CpG dinucleotide is directly seen in read 1 in ABBS samples. FastQC analysis depicting the aggregate base composition of read 1 for ABBS and WGBS. gDNA = genomic DNA. C^m= methylcytosine. **c**, Methylation profiles are bimodal and focused on CpG dinucleotides. Bar plots showing WGBS and ABBS average cytosine methylation level (0-1) versus relative abundance for CpG (CG) dinucleotides, CHG and CHH trinucleotides (H is A, T or C). **d,** ABBS accurately measures cytosine methylation levels. Scatter plot of the methylation levels measured by WGBS and ABBS for all cytosines with a coverage over 25x in both samples. In order to appreciate the influence of extreme values seen in **Fig. 2a**, correlation was calculated for cytosines that have a methylation ranging from 20-80% (Left panel) or for cytosines that have a methylation ranging from 0-100% binned by 5% methylation with equal amount of cytosines per bin (right panel). PCC, Pearson's correlation coefficient with two-tailed $P < 2.2 \times 10^{-16}$ **e,** ABBS signals colocalize with MeDIP-seq coverage and with the presence of 5mC. Genome browser tracks show signal distributions for WGBS, ABBS and MeDIP-seq, as well as methylation levels determined by WGBS and ABBS. **f**, ABBS reads accumulate in genomic elements where 5mC is more prevalent. Metagene analyses of WGBS, ABBS and MeDIP-seq. TSS= Transcription Start Site. TTS= Transcription Termination

Site. **g**, The sensitivity of ABBS surpasses that of WGBS for detection of 5mC. Shown are the relative numbers of 5mC sites (> 50 % methylation) per million reads identified with ABBS (orange) and WGBS (blue), at varying total read counts and with a coverage threshold set at > 25x.
