# Supplementary material for: Anchor-based bisulfite sequencing determines genome-wide DNA methylation
Source: Commun Biol. 2022 Jun 16;5:596. doi: 10.1038/s42003-022-03543-1 (PMC9203462; doi:10.1038/s42003-022-03543-1)
Supplement: Supplementary file 3 — Description of Additional Supplementary Files [file 42003_2022_3543_MOESM3_ESM.pdf]

Description of Additional Supplementary Files

File name: Supplementary Data 1
Description: The source data behind Excel graphs in the paper.
