# Supplementary material for: Anchor-based bisulfite sequencing determines genome-wide DNA methylation
Source: Commun Biol. 2022 Jun 16;5:596. doi: 10.1038/s42003-022-03543-1 (PMC9203462; doi:10.1038/s42003-022-03543-1)
Supplement: Supplementary file 5 — Reporting Summary [file 42003_2022_3543_MOESM5_ESM.pdf]

nature portfolio

Corresponding author(s): Benjamin Delatte

Last updated by author(s): 2022/4/25

Reporting Summary

Nature Portfolio wishes to improve the reproducibility of the work that we publish. This form provides structure for consistency and transparency in reporting. For further information on Nature Portfolio policies, see our Editorial Policies and the Editorial Policy Checklist.

Statistics

For all statistical analyses, confirm that the following items are present in the figure legend, table legend, main text, or Methods section.

n/a	Confirmed	
☐	☒	The exact sample size (*n*) for each experimental group/condition, given as a discrete number and unit of measurement
☐	☒	A statement on whether measurements were taken from distinct samples or whether the same sample was measured repeatedly
☐	☒	The statistical test(s) used AND whether they are one- or two-sided<br>*Only common tests should be described solely by name; describe more complex techniques in the Methods section.*
☒	☐	A description of all covariates tested
☒	☐	A description of any assumptions or corrections, such as tests of normality and adjustment for multiple comparisons
☒	☐	A full description of the statistical parameters including central tendency (e.g. means) or other basic estimates (e.g. regression coefficient) AND variation (e.g. standard deviation) or associated estimates of uncertainty (e.g. confidence intervals)
☐	☒	For null hypothesis testing, the test statistic (e.g. *F*, *t*, *r*) with confidence intervals, effect sizes, degrees of freedom and *P* value noted<br>*Give P values as exact values whenever suitable.*
☒	☐	For Bayesian analysis, information on the choice of priors and Markov chain Monte Carlo settings
☒	☐	For hierarchical and complex designs, identification of the appropriate level for tests and full reporting of outcomes
☐	☒	Estimates of effect sizes (e.g. Cohen's *d*, Pearson's *r*), indicating how they were calculated

Our web collection on statistics for biologists contains articles on many of the points above.

Software and code

Policy information about availability of computer code

Data collection

Raw reads were aligned to the human hg19 or E. coli K12 genomes using Bismark (https://github.com/FelixKrueger/Bismark) [command: $ bismark <genome path> -p 4 --parallel 6 -q --pbat -o <output folder> -1 read1.fastq.gz -2 read2.fastq.gz], and PCR duplicates were removed [command: $ deduplicate_bismark -p --output_dir <output folder> --bam <input bam file>]. A MAPQ filter of 20 was then applied using samtools (https://github.com/samtools/samtools) [command: $ samtools view -h -q 20 <input bam file> -o <output folder bam file>]. Cytosine methylation was measured with Bismark Methylation Extractor [command: $ bismark_methylation_extractor --parallel 10 -p --no_overlap --ignore 7 --ignore_r2 7 --ignore_3prime 5 --ignore_3prime_r2 5 -o <output folder> --report --bedGraph --zero_based --cutoff 1 --CX --remove_spaces --cytosine_report --genome_folder <genome path> <input bam file>]. Next, bam files were sorted and indexed using samtools sort and samtools index. Bigwig coverage files were obtained with deeptools bamcoverage (https://github.com/deeptools/deepTools) [command: $ bamCoverage -b <input bam file> -o <output folder bigwig file> --normalizeUsing RPKM --binSize 5 --numberOfProcessors 16]. The last two columns of the Bismark “zero coverage file” were summed using [command: $ awk 'BEGIN{IFS="\t"}{$5=$5+$6;print $1"\t" $2"\t" $3"\t" $4"\t" $5"\t"}' bismark.zero.cov > bismark.zero.cov.sum.bedgraph]. Chromosome M and non-canonical chromosomes were also removed from the bedgraph files. Cytosines with methylation levels above 50% were then called as methylated [command: $ awk '{ if ($4 > 50) { print } }' <Input bedgraph> > <output bedgraph>]. Cytosines with a coverage above a minimum cutoff were also selected [command: $ awk '{ if ($5 > coverage) { print } }' <Input bedgraph> > <output bedgraph>]. Bam files subsampling was achieved with sabamba (https://lomereiter.github.io/sambamba/) [command: $ sambamba view -h -t 20 -s 0.5 -f bam --subsampling-seed=variable <Input bam file> > -o <output bam file>]. Meta-analyses and metagene analysis were generated using Galaxy interface (https://usegalaxy.org) with computeMatrix and plotHeatmap with defaults parameters (except conversion of missing values to zero). Scatter plots were generated using Galaxy interface with multiBigwigSummary and plotCorrelation (figure 2e), or multiBigwigSummary and RStudio (all other scatter plots), with defaults parameters. CG, CHG and CHH methylation distributions were produced with ViewBS (https://github.com/xie186/ViewBS). Overlaps in figure 2c were calculated with Galaxy Intersect, with 1bp overlap.

Data analysis

Raw reads were aligned to the human hg19 or E. coli K12 genomes using Bismark (https://github.com/FelixKrueger/Bismark) [command: $ bismark <genome path> -p 4 --parallel 6 -q --pbat -o <output folder> -1 read1.fastq.gz -2 read2.fastq.gz], and PCR duplicates were removed [command: $ deduplicate_bismark -p --output_dir <output folder> --bam <input bam file>]. A MAPQ filter of 20 was then applied using

samtools (https://github.com/samtools/samtools) [command: $ samtools view -h -q 20 <input bam file> -o <output folder bam file>]. Cytosine methylation was measured with Bismark Methylation Extractor [command: $ bismark_methylation_extractor --parallel 10 -p --no_overlap --ignore 7 --ignore_r2 7 --ignore_3prime 5 --ignore_3prime_r2 5 -o <output folder> --report --bedGraph --zero_based --cutoff 1 --CX --remove_spaces --cytosine_report --genome_folder <genome path> <input bam file>]. Next, bam files were sorted and indexed using samtools sort and samtools index. Bigwig coverage files were obtained with deeptools bamcoverage (https://github.com/deeptools/deepTools) [command: $ bamCoverage -b <input bam file> -o <output folder bigwig file> --normalizeUsing RPKM --binSize 5 --numberOfProcessors 16]. The last two columns of the Bismark “zero coverage file” were summed using [command: $ awk 'BEGIN{IFS="\t"}{$5=$5+$6;print $1"\t" $2"\t" $3"\t" $4"\t" $5"\t"}' bismark.zero.cov > bismark.zero.cov.sum.bedgraph]. Chromosome M and non-canonical chromosomes were also removed from the bedgraph files. Cytosines with methylation levels above 50% were then called as methylated [command: $ awk '{ if ($4 > 50) { print } }' <Input bedgraph> > <output bedgraph>]. Cytosines with a coverage above a minimum cutoff were also selected [command: $ awk '{ if ($5 > coverage) { print } }' <Input bedgraph> > <output bedgraph>]. Bam files subsampling was achieved with sabamba (https://lomereiter.github.io/sambamba/) [command: $ sambamba view -h -t 20 -s 0.5 -f bam --subsampling-seed=variable <Input bam file> > -o <output bam file>]. Meta-analyses and metagene analysis were generated using Galaxy interface (https://usegalaxy.org) with computeMatrix and plotHeatmap with defaults parameters (except conversion of missing values to zero). Scatter plots were generated using Galaxy interface with multiBigwigSummary and plotCorrelation (figure 2e), or multiBigwigSummary and RStudio (all other scatter plots), with defaults parameters. CG, CHG and CHH methylation distributions were produced with ViewBS (https://github.com/xie186/ViewBS). Overlaps in figure 2c were calculated with Galaxy Intersect, with 1bp overlap.

For manuscripts utilizing custom algorithms or software that are central to the research but not yet described in published literature, software must be made available to editors and reviewers. We strongly encourage code deposition in a community repository (e.g. GitHub). See the Nature Portfolio guidelines for submitting code & software for further information.

Data

Policy information about availability of data

All manuscripts must include a data availability statement. This statement should provide the following information, where applicable:

- Accession codes, unique identifiers, or web links for publicly available datasets
- A description of any restrictions on data availability
- For clinical datasets or third party data, please ensure that the statement adheres to our policy

ABBS and WGBS sequencing data can be found on GEO (GSE180796). MedIP-seq data can be found on GEO (GSM1368906).
RRBS data was downloaded from ENCODE (http://hgdownload.cse.ucsc.edu/goldenPath/hg19/encodeDCC/wgEncodeHaibMethylRr bs/wgEncodeHaibMethylRrbsH1hescHaibSitesRep1.bed.gz).

Field-specific reporting

Please select the one below that is the best fit for your research. If you are not sure, read the appropriate sections before making your selection.

☒ Life sciences ☐ Behavioural & social sciences ☐ Ecological, evolutionary & environmental sciences

For a reference copy of the document with all sections, see nature.com/documents/nr-reporting-summary-flat.pdf

Life sciences study design

All studies must disclose on these points even when the disclosure is negative.

Sample size	For bacteria data, 2 clones and two library preparation kits were used, for human K562 cells, 2 replicates with 2 library preparation kits were performed.
Data exclusions	No data points were excluded.
Replication	Reproducibility was confirmed between replicates.
Randomization	No randomization was involved in this manuscript, N/A.
Blinding	N/A.

Reporting for specific materials, systems and methods

We require information from authors about some types of materials, experimental systems and methods used in many studies. Here, indicate whether each material, system or method listed is relevant to your study. If you are not sure if a list item applies to your research, read the appropriate section before selecting a response.

Materials & experimental systems

n/a	Involved in the study
☒	☐ Antibodies
☐	☒ Eukaryotic cell lines
☒	☐ Palaeontology and archaeology
☒	☐ Animals and other organisms
☒	☐ Human research participants
☒	☐ Clinical data
☒	☐ Dual use research of concern

Methods

n/a	Involved in the study
☒	☐ ChIP-seq
☒	☐ Flow cytometry
☒	☐ MRI-based neuroimaging

Eukaryotic cell lines

Policy information about cell lines

Cell line source(s)	K562.
Authentication	Bought from ATCC, no further identification
Mycoplasma contamination	No contamination was detected.
Commonly misidentified lines (See ICLAC register)	N/A.
